# A Novel Mutation of the *FBN1* Gene in a Chinese Family With Marfan Syndrome and Unanticipated Discoveries of Family Members

**DOI:** 10.1155/genr/7350256

**Published:** 2026-09-22

**Authors:** Hang Shi, Xuejian Han, Shukai Xing, Yuetong Zhao, Yanyan Wang, Mengtao Yun, Yang Lou, Xiaobo Cui, Baichun Wang, Xiangning Meng

**Affiliations:** ^1^ Department of Medical Genetics, School of Basic Medical Sciences, Harbin Medical University, Harbin 150081, China, hrbmu.edu.cn; ^2^ Key Laboratory of Preservation of Human Genetic Resources and Disease Control in China, Harbin Medical University, Ministry of Education, Harbin 150081, China, meb.gov.tr; ^3^ Department of Cardiovascular Surgery, The Fourth Affiliated Hospital of Harbin Medical University, Harbin 150001, China, hrbmu.edu.cn; ^4^ Teaching Experiment Center of Biotechnology, School of Basic Medical Sciences, Harbin Medical University, Harbin 150081, China, hrbmu.edu.cn; ^5^ Teaching Center of Morphology, School of Basic Medical Sciences, Harbin Medical University, Harbin 150081, China, hrbmu.edu.cn

**Keywords:** *FBN1* gene, gene mutation, Marfan syndrome, whole-exome sequencing

## Abstract

Marfan syndrome (MFS) is an autosomal dominant connective tissue disorder caused by pathogenic variants in the *FBN1* gene. Using whole‐exome sequencing (WES) in a Chinese MFS family, we identified a novel heterozygous nonsense variant *FBN1*:c.1415dup;p.(Tyr472Ter), which cosegregates with the disease phenotype and results in protein truncation. Immunohistochemical analysis confirmed markedly reduced fibrillin‐1 expression in the aortic wall of variant carriers, and this variant was classified as pathogenic. Unexpectedly, the proband’s younger sister harbors three independent pathogenic variants responsible for MFS, familial hypercholesterolemia (FH), and oculopharyngeal muscular dystrophy (OPMD). Further transcript validation and functional assays demonstrated that the *LDLR*:c.1987+1G > A;p.Arg662_Gly663insAspLys splice‐site mutation generates a stable in‐frame insertion transcript, impairing protein structure and function. Supported by robust functional evidence and familial cosegregation, this *LDLR* variant was upgraded from a variant of uncertain significance to pathogenic. We provided formal genetic counseling, long‐term clinical follow‐up, and multidisciplinary surveillance to all affected family members, with particularly comprehensive management for the proband’s sister. This study expands the mutational spectrum of *FBN1*, establishes functional evidence for the pathogenicity of the *LDLR* variant, and represents the first report of a pediatric individual carrying three coexisting pathogenic variants for rare diseases. Our findings provide critical insights for molecular diagnosis, genetic counseling, and clinical risk stratification in affected families.

## 1. Introduction

Marfan syndrome (MFS) is an autosomal dominant connective tissue disorder with a prevalence of 1–2 per 10,000 individuals [1]. It predominantly affects the cardiovascular, ocular, and skeletal systems, manifesting as aortic abnormalities, valvular defects, lens dislocation, arachnodactyly, and scoliosis [2, 3]. The etiology of MFS is linked to mutations in the *FBN1* gene (located at 15q21.1, comprising 65 exons) [4], which encodes fibrillin‐1, a crucial component of extracellular microfibrils [5].

Familial hypercholesterolemia (FH) is an autosomal dominant lipid disorder with a prevalence of 1 in 500 [6], characterized by elevated cholesterol levels, xanthomas, and premature cardiovascular disease [7]. Approximately 90% of FH cases are attributed to mutations in the *LDLR* gene, which impair low‐density lipoprotein (LDL) clearance [8–10].

Oculopharyngeal muscular dystrophy (OPMD) is a late‐onset myopathy with an incidence of 1 in 100,000 individuals. It typically presents after the age of 45, with symptoms including ptosis, dysphagia, and limb weakness [11]. OPMD is caused by a guanine–cytosine‐nucleotide (GCN) repeat expansion in exon 1 of the *PABPN1* gene.

In this study, whole‐exome sequencing (WES) was conducted on a Chinese family affected by MFS. A mutation in the *FBN1* gene, specifically c.1415dup, was identified in the proband, as well as in his father and sister. This particular mutation has previously been documented only in a patient with MASS syndrome [12] and not in families with MFS. Notably, the proband’s sister was found to carry three pathogenic variants associated with MFS, FH, and OPMD, which were inherited from both parents. To the best of our knowledge, this is the first documented case of such a genetic finding.

## 2. Materials and Methods

### 2.1. Research Object

**FIGURE 1:**
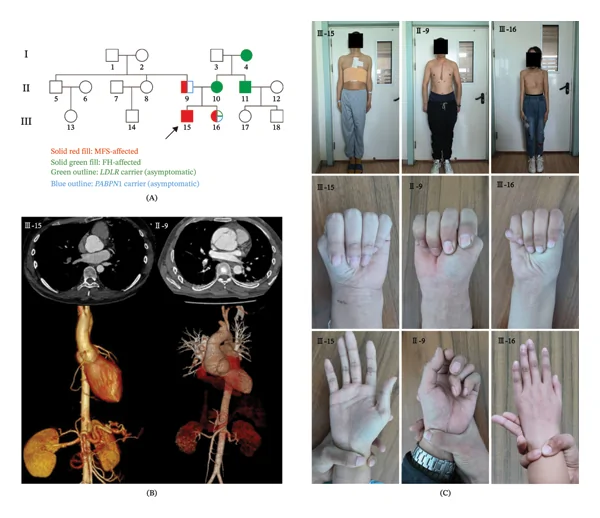
Pedigree, imaging, and physical examination findings of the multi‐gene pedigree. (A) Three‐generation pedigree. Solid red symbols: MFS‐affected individuals; solid green symbols: FH patients. Green‐outlined symbols: asymptomatic *LDLR* carriers; blue‐outlined symbols: asymptomatic *PABPN1* carriers. Unfilled symbols represent unaffected relatives; the black arrow indicates the proband (III‐15). (B) Reconstructed aortic CTA scans of the proband (III‐15) and his father (II‐9). (C) Clinical photos of III‐15, II‐9, and III‐16 showing body habitus, thoracic deformity, wrist sign, and thumb sign.

A 17‐year‐old male patient, referred to as the proband, was admitted to the Cardiovascular Surgery Department of the Fourth Affiliated Hospital of Harbin Medical University in February 2023 due to aneurysmal dilation of the aortic root and was subsequently selected as the subject of this study. The investigation also included three generations of his family, comprising his father, mother, sister, grandparents, aunt, uncle, cousins, maternal grandmother, and maternal uncle, as illustrated in Figure 1A. The patient received a clinical diagnosis of MFS based on clinical manifestations and imaging examinations, including echocardiography and aortic CT angiography, in accordance with the 2010 Revised Ghent criteria for MFS [13]. This diagnosis was further supported by the 2017 Chinese Expert Consensus on the Diagnosis and Treatment of Aortic Dissection and Aneurysm [14] and the 2014 European Society of Cardiology (ESC) Guidelines on the Diagnosis and Treatment of Aortic Diseases [15]. Additionally, laboratory data concerning blood lipid profiles, including total cholesterol, triglycerides, and low‐density lipoprotein cholesterol (LDL‐C), were collected from the proband’s mother, sister, maternal grandmother, and maternal uncle. Normal ascending aorta tissue samples were obtained from an organ donor who had been brain‐dead as a result of an accident. This study adhered to the Declaration of Helsinki and received approval from the Ethics Committee of the Fourth Affiliated Hospital of Harbin Medical University (Approval No. 2023‐YXLLSC‐18). The informed consent forms were signed by the patient and his family members.

### 2.2. Genomic DNA Extraction

2 mL of peripheral blood from the proband and family members was collected. For each sample, 200 μL of blood was taken to extract genomic DNA using a whole blood DNA extraction kit (TIANGEN, China). Quantification was performed using a NanoDrop 2000c spectrophotometer, and the quality of the extracted DNA was assessed using 1% agarose gel electrophoresis.

### 2.3. WES

Library target gene capture and high‐throughput sequencing were carried out using WES (Beijing Berry Genomics Co, Ltd) to capture single‐nucleotide variants within the exon regions and splice junction boundaries (5 bp range) of the subjects, as well as insertions/deletions within 50 bp of the exon regions. The captured regions were then subjected to paired‐end sequencing on the Illumina HiSeq X 10 high‐throughput sequencing platform, with a read length of 150 bp.

### 2.4. RNA Extraction

RNA was isolated from separated peripheral blood mononuclear cells (PBMCs) of blood samples using the Trizol method. RNA concentration and purity were assessed using a NanoDrop 2000c spectrophotometer.

### 2.5. RT‐PCR

RNA extracted from PBMCs was reverse‐transcribed into cDNA. Three primer sets were designed for validation, including genomic primers for *FBN1* and *LDLR*, and cDNA primers for *LDLR* transcript analysis, and are provided in Supporting Table 1. Polymerase chain reaction (PCR) was performed at 95°C for 3 min, followed by 40 cycles of 95°C for 30 s, annealing for 30 s, and 72°C for 30 s, with a final extension at 72°C for 5 min. Products were examined by 2% agarose gel electrophoresis.

### 2.6. Sanger Sequencing

First, synonymous mutations of the *FBN1* and *LDLR* genes that did not affect splicing, intronic mutations without literature reports, and variants with a frequency of ≥ 2% in normal populations were excluded. Literature reports and software prediction results were also included. Further screening was then carried out based on the inheritance pattern of the genes, taking into account whether the genetic inheritance model was consistent with pathogenic patterns, and combined with clinical phenotype mutations to further refine the selection of mutations. Sequencing was performed using the forward primer, and the amplified samples were sent to Shanghai Sangon Biotech Co., Ltd. to test the candidate variants obtained from high‐throughput sequencing.

### 2.7. Capillary Electrophoresis

The capillary with an inner diameter of 50 μm was cut to an appropriate length and filled with electrophoresis buffer. The sample was injected into one end of the capillary at a voltage of 20 kV and a constant temperature of 25°C. Under the action of the electric field, the sample migrated in the capillary, and electrophoresis analysis was then initiated. During the analysis, the stability of the detection signal and the symmetry of the peak shape were monitored to evaluate the separation efficiency, ensuring accurate determination of *PABPN1* fragment sizes.

### 2.8. Protein Structure Prediction

Amino acid sequence prediction was performed for the wild‐type and mutant *FBN1* and *LDLR* genes, and comparative three‐dimensional structural models of the wild‐type and mutant proteins were constructed using Swiss‐Model (https://swissmodel.expasy.org/, updated 2023).

### 2.9. Detection of Species Conservation

The conservation of the target gene/sequence among different species was detected using the National Center for Biotechnology Information (NCBI) database (https://www.ncbi.nlm.nih.gov/). The target sequence and its homologous sequences from different species were retrieved from the NCBI Gene database in FASTA format, and homologous alignment was performed using the BLAST tool.

### 2.10. Immunohistochemistry

After deparaffinization, sections were treated with H_2_O_2_ for 10 min and subjected to antigen retrieval in citrate buffer (pH 6.0) for 4 min. After cooling, sections were blocked with 1% bovine serum albumin (BSA) for 1 h, and then incubated with anti‐fibrillin‐1 antibody (ABclonal, China) overnight at 4°C. Following PBS washes, sections were incubated with biotinylated rabbit secondary antibody (Zhongshan Jinqiao, China) for 20 min. Staining was visualized using a DAB kit (Zhongshan Jinqiao, China) according to the manufacturer’s protocol.

### 2.11. Clinical Notes on Functional Prediction and Variation

Functional and conservation analyses of the *FBN1* gene variants were conducted using software such as PolyPhen‐2 (v2.2.2), SIFT (v6.2.1), MutationTaster (v2021), and REVEL (v1.3). The classification of genetic variants was performed according to the standards and guidelines of the American College of Medical Genetics and Genomics (ACMG) [16].

## 3. Results

### 3.1. Clinical Study Results

The proband (III‐15), a 17‐year‐old male, presented with a complaint of sudden chest pain for three days and was diagnosed with aortic root aneurysmal dilation (Figure 1B). At the time of admission, II‐9 was 178 cm in height and weighed 75 kg, with mild scoliosis. The proband’s sister (III‐16), 8 years old, is tall and thin, with arachnodactyly, severe myopia, pectus excavatum, scoliosis, and other signs and symptoms associated with MFS (Figure 1C). Table 1 presents the systematic scoring for the diagnosis of MFS in the proband (III‐15), his father (II‐9), and his sister (III‐16). It should be noted that other family members did not display any signs or symptoms related to MFS.

**TABLE 1:** Systematic scoring for the diagnosis of Marfan syndrome family members.

Characterization	III‐15	II‐9	III‐16
Wrist and thumb sign (3)			3
Wrist or thumb sign (1)			
Pectus carinatum deformity (2)			
Pectus excavatum or chest asymmetry (1)	1		1
Hindfoot deformity (2)			1
Plain pes planus (1)	1	1	
Pneumothorax (2)	2		
Dural ectasia (2)			
Protrusio acetabuli (2)			
Reduced US/LS and increased arm/height and no severe scoliosis (1)	1		
Scoliosis or thoracolumbar kyphosis (1)	1	1	1
Reduced elbow extension (1)	1		1
Facial features (3/5): dolichocephaly, enophthalmos, down‐slanting palpebral fissures, malar hypoplasia, retrognathia (1)			
Skin striae (1)	1		
Myopia > 3 diopters (1)	1		1
Mitral valve prolapse: all types (1)	1		
Total	10	2	8

*Note:* Maximum total: 20 points; score ≥ 7 indicates systemic involvement.

Abbreviation: US/LS = upper segment/lower segment ratio.

As presented in Table 2, three members from the proband’s maternal family (I‐4, II‐10, and II‐11) exhibited increased total cholesterol and LDL cholesterol levels above the normal reference ranges, with II‐10 showing the most severe dyslipidemia. Conversely, individual III‐16 had completely normal lipid profiles. Triglyceride levels remained normal in all subjects, while reduced HDL‐cholesterol was noted in II‐11.

**TABLE 2:** Lipid profiles of familial hypercholesterolemia members.

Lipid parameters	I‐4	II‐10	II‐11	III‐16
Total cholesterol (mmol/L) (ref: < 5.18)	5.48	6.70	5.54	4.33
Triglyceride (mmol/L) (ref: 0.56–1.70)	0.57	0.95	1.31	1.46
HDL cholesterol (mmol/L) (ref: 1.04–1.55)	1.49	1.17	0.70	1.31
LDL cholesterol (mmol/L) (ref: < 3.37)	3.91	4.35	3.73	1.89

*Note:* ref, reference range.

Abbreviations: HDL = high‐density lipoprotein, LDL = low‐density lipoprotein.

### 3.2. WES Results

WES identified a heterozygous nonsense mutation in the *FBN1* gene (NM_000138.5:c.1415dup) in the proband (III‐15), his father (II‐9), and his sister (III‐16). This mutation is located in exon 12 and was absent in other family members and normal population databases. No additional pathogenic variants associated with aortic diseases were detected. Additionally, a splice‐site mutation in the *LDLR* gene (NM_000527.5:c.1987+1G > A) was identified in the proband’s sister (III‐16). Subsequent family segregation analysis confirmed the presence of this mutation in the proband’s mother, uncle, and maternal grandmother, indicating a pattern of maternal inheritance. Moreover, both the proband’s father (II‐9) and sister (III‐16) were found to carry a GCN trinucleotide repeat expansion (≥ 11 repeats) in exon 1 of the *PABPN1* gene, which is associated with OPMD (Table 3).

**TABLE 3:** Pathogenic variant information of the pedigree.

Subject	Gene	Mutation location	Gene subregion	HGVS	Mutation type	Heterozygosity
II‐9 III‐15 III‐16	*FBN1*	chr15: 48515439‐48515439	Exon 12	NM_000138.5:c.1415dup	Stop gained	Heterozygous

I‐4 II‐10 II‐11 III‐16	*LDLR*	chr19: 11120234‐11120234	Intron 13	NM_000527.5: c.1987+1G > A	Splice donor variant	Heterozygous

II‐9 III‐16	*PABPN1*	chr14: 23321472‐23321502	Exon 1	NM_004643.4: c.4_6GCN[10]; 4_6GCN[11]	Dynamic mutation	Heterozygous

### 3.3. Sanger Sequencing and Capillary Electrophoresis Results

**FIGURE 2:**
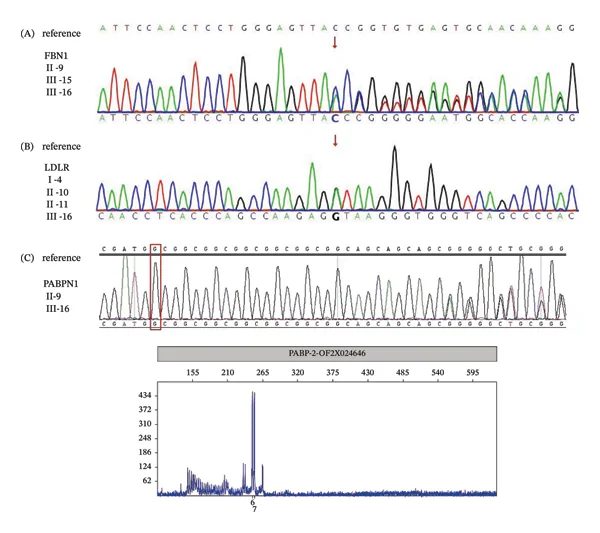
Results of mutation gene detection in an MFS family. (A) Sanger sequencing results of *FBN1* mutation. The sequence chromatogram shows a heterozygous nonsense mutation (NM_000138.5: c.1415dup) in *FBN1*. The red arrow indicates the mutation site. (B) Sanger sequencing results of *LDLR* mutation. The sequence chromatogram shows a heterozygous splice site mutation (NM_000527.5: c.1987+1G > A). The red arrow indicates the mutation site. (C) Electrophoretic results of *PABPN1* mutation. The capillary electrophoresis chromatogram shows the *PABPN1*:c.4_6GCN[10]; 4_6GCN[11] mutation, with a sharp main peak for the normal fragment and a clustered, low, spiky claw‐shaped peak for the expanded repetitive sequence.

DNA was extracted from peripheral blood and subjected to PCR amplification, followed by Sanger sequencing. It was determined that the proband’s father (II‐9) and sister (III‐16) both exhibited the same *FBN1*:c.1415dup mutation as the proband (III‐15), which is a nonsense mutation. In contrast, unaffected individuals in the family with no clinical phenotypes did not carry this mutation (Figure 2A). The proband’s sister (III‐16), mother (II‐10), uncle (II‐11), and grandmother (I‐4) were all found to be carriers of the same mutation (*LDLR*:c.1987+1G > A), which is a splice mutation (Figure 2B). Capillary electrophoresis demonstrated that the proband’s father (II‐9) and sister (III‐16) exhibited an aberrant amplification of a single (guanine–cytosine–guanine [GCG]) repeat within the *PABPN1* gene exon, indicative of heterozygosity for GCN10/GCN11 (Figure 2C).

### 3.4. Protein Structural Analysis of FBN1 Variants

**FIGURE 3:**
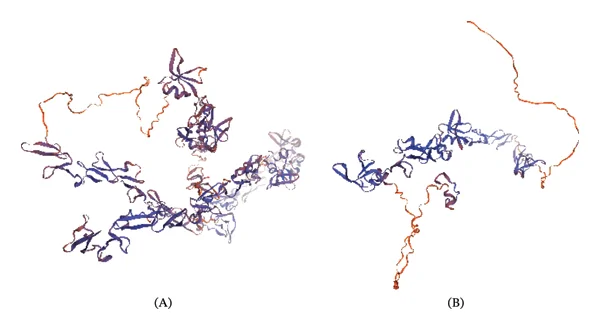
The Swiss model simulates the three‐dimensional structure of wild‐type and mutant *FBN1* protein. (A)Wild‐type; (B) mutant.

The Swiss‐Model server was used to generate three‐dimensional structural models of mutant fibrillin‐1 protein from the proband (III‐15), his father (II‐9), and his sister (III‐16). The wild‐type *FBN1* gene encodes a precursor protein comprising 2871 amino acids. In contrast, the pathogenic variant *FBN1*:c.1415dup;p. (Tyr472Ter) yields a truncated peptide of only 471 amino acids, leading to the loss of most canonical structural domains of fibrillin‐1. This frameshift mutation drastically remodels the overall protein conformation, severely impairs physiological function, and abolishes the production of normal full‐length fibrillin‐1. Comparative structural models of wild‐type and mutant fibrillin‐1 are displayed in Figure 3.

### 3.5. Transcript Validation and Protein Structural Analysis of LDLR Variants

**FIGURE 4:**
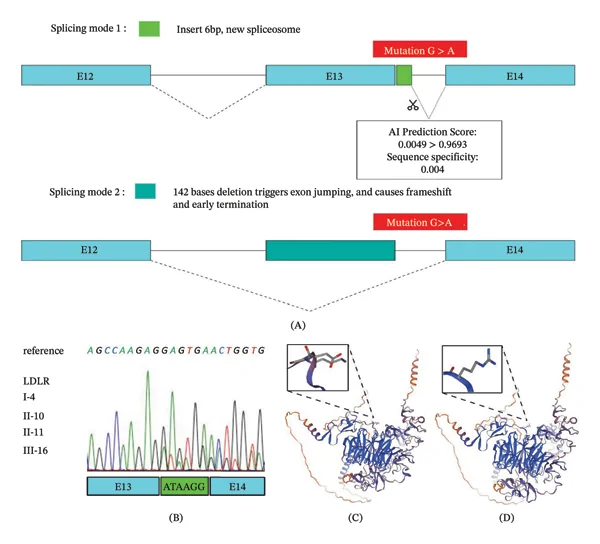
Functional characterization of the *LDLR*:c.1987+1G > A variant. (A) Wild‐type reading frame, mutant 1 inserting 6 bp and causing a new spliceosome, and mutant 2 causing a 142 bp exon skipping, frameshift alteration, and premature termination. (B) Sanger sequencing results demonstrate a heterozygous mutation characterized by a 6‐bp insertion between Exons 13 and 14 in I‐4, II‐10, II‐11 and III‐16. (C) Predicted three‐dimensional structure of wild‐type *LDLR*. (D) Predicted three‐dimensional structure of mutant 1.

Three‐dimensional structural models of *LDLR* proteins from the proband’s mother (II‐10), sister (III‐16), maternal grandmother (I‐4), and maternal uncle (II‐11) were developed. Bioinformatic analyses predicted that the *LDLR*:c.1987+1G > A splice mutation would result in the production of two aberrant splicing isoforms (Figure 4A). However, subsequent Sanger sequencing of RNA transcripts confirmed the in vivo production of only Mutant 1 (Figure 4B). This mutation involves a 6‐base‐pair in‐frame insertion that introduces two additional amino acids, aspartic acid and lysine, thereby altering the length of the *LDLR* protein and causing conformational disruptions in the *LDLR*, compromising its native structure and biological function, and impeding the normal synthesis of *LDLR* precursors. Mutant 2 was not detected. Structural models of both the wild‐type and mutant 1 forms (*LDLR*:c.1987+1G > A;p.Arg662_Gly663insAspLys) are illustrated in Figures 4C and D.

### 3.6. Immunohistochemical Results

**FIGURE 5:**
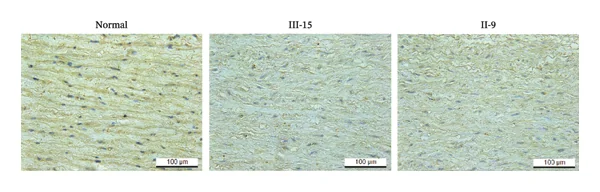
The expression of *FBN1* in the aortic wall of patients with MFS and normal control. Immunohistochemical analysis shows the levels of fibrillin‐1 in the aortic specimens of normal control, proband (III‐15), and his father (II‐9). Scale bar = 100 μm.

Aortic specimens were obtained from the proband (III‐15) and his father (II‐9) through surgical procedures and subjected to immunohistochemical analysis following paraffin embedding. Given the limited number of samples, statistical analysis was not feasible. However, immunohistochemical results indicated that the expression levels of *FBN1*‐encoded fibrillin‐1 in the aortic specimens of the proband (III‐15) and his father (II‐9) were significantly lower than those of normal control patients (Figure 5). These results are in accordance with the findings of exon sequencing, which indicated that the mutations resulted in impaired protein expression, thereby confirming the diagnosis of MFS in the two patients.

### 3.7. Species Conservation Analysis

**FIGURE 6:**
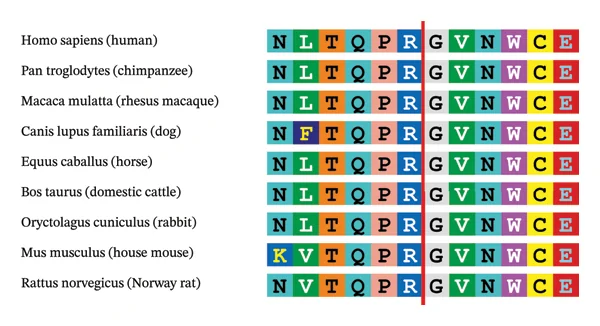
Cross‐species alignment of LDLR orthologs in nine mammals. The vertical red line marks the insertion region of p.Arg662_Gly663insAspLys, whose residues are highly conserved among all species, with minor N‐terminal substitutions only found in dog, mouse, and rat.

Cross‐species multiple sequence alignment of orthologous proteins from nine mammalian species revealed extremely high evolutionary conservation of the target peptide region (Figure 6). The amino acid residues flanking the insertion variant *LDLR*:p.Arg662_Gly663insAspLys are nearly invariant across all vertebrate orthologs; sparse amino acid substitutions were only detected at two N‐terminal positions in dog, mouse, and rat. The strict retention of this sequence throughout mammalian evolution highlights that this conserved peptide motif performs irreplaceable structural and physiological functions.

### 3.8. Pathogenicity Analysis

The *FBN1*:c.1415dup;p.(Tyr472Ter) variant was classified as pathogenic according to ACMG guidelines. The evidence included: (1) PVS1: introduction of a premature stop codon leading to disrupted protein function; (2) PS1: identical amino acid alteration as previously confirmed pathogenic variants at the same residue; (3) PM2: extremely low allelic frequency in population databases; and (4) PP1: cosegregation with disease phenotype in multiple affected family members verified by Sanger sequencing.

The *LDLR*:c.1987+1G > A;p.Arg662_Gly663insAspLys variant was classified as pathogenic according to ACMG guidelines. The evidence included: (1) PS4: significantly higher prevalence in affected individuals compared with controls; (2) PM1: located in a known mutational hotspot and critical splice donor site of *LDLR*; (3) PM2: extremely low allelic frequency in population databases; (4) PM4: confirmed in‐frame insertion altering the *LDLR* protein sequence; (5) PP1: cosegregation with FH phenotype in multiple affected family members; and (6) PP3: multiple computational predictions supporting a deleterious effect on splicing and protein function.

The *PABPN1*:c.4_6GCN[10];4_6GCN[11] was classified as pathogenic according to ACMG standards for repeat expansion disorders. The evidence included the following: (1) the repeat number exceeded the well‐established pathogenic threshold (≥ 11 GCN repeats) for OPMD; (2) the expansion segregated with the disease risk in the family and was inherited from the father; and (3) the genotype was consistent with the autosomal dominant inheritance pattern and late‐onset clinical phenotype of OPMD.

## 4. Discussion

MFS is an autosomal dominant disorder mainly caused by *FBN1* mutations. According to the 2010 Ghent criteria, *FBN1* mutation detection is critical for MFS diagnosis [13]. In this study, the proband presented tall stature, arachnodactyly, scoliosis, high myopia, skin striae, and marked aortic root dilation, with a systemic score ≥ 7, leading to a clinical MFS diagnosis. The proband’s father had a history of aortic dissection without obvious MFS features, while his sister had a systemic score ≥ 7 but incomplete aortic evaluation. Genetic testing confirmed that both the father and sister carried the pathogenic mutation.

The proband carries the *FBN1* gene mutation:c.1415dup. This mutation is located in exon 12 and results from the insertion of a base, leading to a nonsense mutation that generates a shortened RNA and an abnormal protein. Further protein structural analysis reveals that the normal *FBN1* gene encodes a protein with 2871 amino acids, while the fibrillin‐1 encoded by the c.1415dup mutation consists of only 471 amino acids. This results in the loss of the majority of the normal structure of fibrillin‐1, which is completely altered in its conformation and therefore unable to convert into normal fibrillin‐1. According to the evaluation guidelines established by ACMG, this locus is classified as a pathogenic mutation. The mutation was initially reported by Laura Muiño‐Mosquera et al. in 2018, who associated it with MASS syndrome. MASS syndrome is a milder form of an *FBN1*‐related connective tissue disorder, marked by mitral valve prolapse, mild aortic root enlargement, skeletal issues, and skin changes, without severe aortic complications or lens dislocation [17]. However, the proband (III‐15) in this study exhibited significant aortic root aneurysmal dilation, a history of pneumothorax, severe skeletal issues, and a systemic score ≥ 7, aligning with classic MFS rather than MASS. Although the same *FBN1* mutation is linked to MASS, our findings suggest it can also lead to severe MFS, indicating variable expressivity. This phenotypic variation may be due to genetic modifiers, epigenetic factors, or other individual differences [18].

Fibrillin‐1 is a 2871‐amino acid glycoprotein containing multiple functional domains. It is broadly expressed in elastic tissues, including cartilage, cornea, heart valves, and blood vessels. Dysfunction of fibrillin‐1 leads to pathological changes in these tissues, resulting in the characteristic skeletal, ocular, and cardiovascular phenotypes of MFS. To date, more than 2000 *FBN1* mutations have been reported in MFS, with no obvious mutation hotspots [19]. The main mutation types include frameshift, missense, and nonsense mutations. Nonsense mutations account for approximately 12% of all *FBN1* mutations and cause fibrillin‐1 haploinsufficiency (HI‐*FBN1*), which is a major mechanism of pathogenesis [18]. Previous studies have shown that HI‐*FBN1* mutations are associated with shorter event‐free survival, more severe cardiovascular complications, and higher risks of aortic dissection and mortality [20–22]. Therefore, our genetic findings confirm the diagnosis of the proband’s clinically atypical father, help avoid misdiagnosis, and enable early surveillance and optimized intervention for at‐risk family members, especially the younger sister, thereby delaying acute cardiovascular events and improving prognosis.

Genetic testing showed that the proband’s sister and three maternal relatives carried a pathogenic *LDLR* splice‐site mutation inherited from their mother, which is associated with autosomal dominant FH. *LDLR*:c.1987+1G > A is located at the exon 13/intron 13 boundary and represents a mutational hotspot of the gene. This variant has been previously detected in hypercholesterolemia cohorts [23]. Bioinformatic analysis predicted two aberrant transcripts, and RNA validation confirmed that only Mutant 1 with a 6‐bp in‐frame insertion is stably expressed, resulting in an extended protein sequence, altered conformation, and impaired receptor function. Multi‐species alignment revealed that the affected splice site and adjacent sequence are highly conserved across mammals, indicating the functional importance of this region. In this family, the proband’s mother (II‐10), maternal uncle (II‐11), and maternal grandmother (I‐4) all carried the same mutation and exhibited markedly elevated total cholesterol and LDL‐C levels, consistent with an autosomal dominant inheritance pattern. Although blood lipid levels can be influenced by environmental factors, the persistent and severe hypercholesterolemia in all mutation carriers supports a primary genetic etiology. Notably, this variant was previously classified as a variant of uncertain significance in public databases and literature; based on functional validation, familial cosegregation, and comprehensive analysis, we upgraded its classification to pathogenic. Collectively, *LDLR*:c.1987+1G > A is a pathogenic variant responsible for FH in this family. Long‐standing hypercholesterolemia is a major risk factor for cardiovascular complications [24]. It is therefore crucial for family members carrying the mutation to adopt a healthy lifestyle and effectively manage cardiovascular risk factors, including blood pressure, lipid levels, and blood glucose, in order to mitigate the progression of vascular disease. While statins represent the primary treatment, alternatives such as PCSK9 inhibitors may be necessary for patients intolerant to statins or not meeting treatment goals [6]. Additionally, gene therapy and new drugs offer promising options for managing FH.

OPMD is associated with a dynamic mutation in the GCN triplet repeat in exon 1 of the *PABPN1* gene on chromosome 14q11.2–14q13 [25]. The wild‐type *PABPN1* gene contains 10 repeats of GCN, whereas individuals affected by the mutation have 11–18 repeats [26]. Previous studies have shown that repeat copy number correlates with age at onset and disease severity, but not with clinical manifestations during childhood or early adulthood [25, 26]. Here, the proband’s father (II‐9) and 8‐year‐old sister (III‐16) both carry a heterozygous *PABPN1*:c.4_6GCN[10];4_6GCN[11] genotype. Heterozygous GCN11 expansions have been firmly established as pathogenic [27, 28]. Notably, both II‐9 and III‐16 remain asymptomatic, with no ptosis, dysphagia, or proximal weakness. This is fully consistent with the natural history of OPMD, a late‐onset autosomal dominant myopathy that almost never presents before age 40 years, with typical onset at 45–50 years [25]. The absence of clinical features at their current ages thus does not conflict with pathogenicity but reflects the typical age‐dependent penetrance and late onset of OPMD. Both are molecularly confirmed pathogenic variant carriers, not clinically affected individuals. Long‐term prospective follow‐up is warranted.

The proband’s sister (III‐16) carries pathogenic variants associated with three rare disorders: MFS, FH, and OPMD. Mechanistically, the three pathogenic variants follow independent inheritance patterns: the *FBN1* and *PABPN1* variants were transmitted from the father, whereas the *LDLR* splice‐site mutation was inherited from the mother. No direct functional interaction or synergistic pathogenic mechanism among the three mutant genes is known at present. However, from a clinical perspective, it is noteworthy that the maternal lineage has exhibited elevated cholesterol levels. The coexistence of MFS‐related aortic disease and FH‐related progressive atherosclerosis may confer long‐term synergistic risks to the cardiovascular system, warranting lifelong multidisciplinary monitoring. Formal genetic counseling was provided to the patient and her legal guardians, including the clinical implications of the variants, disease risks, inheritance pattern, and long‐term follow‐up strategy. Long‐term clinical follow‐up, personalized health guidance, and standardized follow‐up archive management were performed. She currently presents with thoracic and skeletal malformations related to MFS. Regular evaluations of pectus excavatum and scoliosis were conducted by orthopedic and cardiothoracic surgeons, and conservative interventions including orthoses and vacuum suction cups were administered accordingly. Ophthalmological assessments were performed periodically to monitor refractive status and lens position. Aortic sinus surveillance was carried out by echocardiography, showing no dilatation at present. Although pharmacological intervention is not yet required, strenuous exercise, competitive sports, and weight training should be avoided to reduce the risk of aortic dilatation. Standard medical therapy will be initiated promptly if aortic sinus dilatation develops in the future. For FH, lipid profiling will be performed every 2–3 years. Given the late‐onset characteristic of OPMD, long‐term clinical follow‐up and observation are recommended.

## 5. Conclusions

In this study, we identified a novel pathogenic *FBN1*:c.1415dup;p.(Tyr472Ter) in a family with MFS, characterized its phenotype, and expanded the *FBN1* mutational spectrum for improved diagnosis and genetic counseling. We also functionally validated *LDLR*:c.1987+1G > A;p.Arg662_Gly663insAspLys and upgraded it to pathogenic, strengthening molecular diagnosis and clinical management for FH. Furthermore, we report the first case of concurrent pathogenic variants for three rare diseases (MFS, FH, and OPMD), providing valuable insights for the molecular diagnosis, genetic counseling, and multidisciplinary management of complex comorbid rare diseases.

## 6. Limitations

Several limitations of this study warrant mention. First, as a single family report, the small sample size restricts the statistical power and generalizability of the genotype–phenotype correlation for the *FBN1*:c.1415dup variant. Second, functional investigations remain preliminary; in‐depth mechanistic studies, including luciferase assays, cellular functional validation, and animal models, were not performed to define the molecular consequences of the *FBN1* and *LDLR* mutations.

## Author Contributions

Hang Shi analyzed the data and participated in manuscript writing. Hang Shi, Mengtao Yun, and Yang Lou collected the family blood samples and the clinical data. Xiangning Meng and Hang Shi performed bioinformatic analysis. Hang Shi, Shukai Xing, and Yanyan Wang finished the laboratory experiments. Xuejian Han, Yuetong Zhao, and Xiaobo Cui helped in manuscript preparation and editing. Xiangning Meng and Baichun Wang designed and modified the manuscript.

## Funding

This research was funded by Internal Fund from the Fourth Affiliated Hospital of Harbin Medical University (HYDSYYZ201605), Horizontal Project of Harbin Medical University (No. 22992230031), Graduate Innovation Fund from Harbin Medical University (YJSCX2023‐282HYD), and Natural Science Foundation of Heilongjiang Province, China (No. ZL2024H010).

## Disclosure

All authors have read and agreed to the published version of the manuscript.

## Ethics Statement

The study was conducted according to the guidelines of the Declaration of Helsinki and approved by the Ethics Committee of the Fourth Affiliated Hospital of Harbin Medical University (Approval No. 2023‐YXLLSC‐18). Approval date (15 June 2023).

## Consent

Written informed consent has been obtained from the patient(s) to publish this paper.

## Conflicts of Interest

The authors declare no conflicts of interest.

## Supporting Information

Additional supporting information can be found online in the Supporting Information section.

## Supporting information


**Supporting Information** Supporting Table 1. Primer sequences for RT‐PCR experiments.

## Data Availability

The data produced in this research are presented in this manuscript and will be publicly available.
