# Supplementary material for: A Novel Mutation of the FBN1 Gene in a Chinese Family With Marfan Syndrome and Unanticipated Discoveries of Family Members
Source: Genet Res (Camb). 2026 Sep 22;2026:7350256. doi: 10.1155/genr/7350256 (PMC13598443; doi:10.1155/genr/7350256)
Supplement: Supplementary file 1 [file GENR-2026-7350256-s001.docx]

**Supplementary Table 1.** Primer sequences for RT-PCR experiments

| Primer Name | Sequence (5'-3') | Annealing Temp (°C) |
| --- | --- | --- |
| *FBN1* | F:TCCCCAGTGCTCACAGTGC  R:AAACCAGTAGAGTCAAGGAACAGAAT | 60 |
| *LDLR* | F: CAGTGTTTAACGGGATTTGTCAT  R: AGAAGCGGAGTCAGGGCA | 59 |
| *LDLR*(cDNA) | F: CCGGAAGACCATCTTGGAGG  R:GTAAACTTGGGCGAGTGGGG | 63 |
